# Retrospective assessment of low-dose and low-contrast agent protocols for photon-counting CT angiography of the aorta

**DOI:** 10.1007/s10554-026-03757-y

**Published:** 2026-06-19

**Authors:** Jan-Lucca Hennes, Kristina Krompaß, Henner Huflage, Pauline Pannenbecker, Ramin Saam Dazeh, Jan-Peter Grunz, Viktor Hartung, Thorsten Alexander Bley, Philipp Gruschwitz

**Affiliations:** Department of Diagnostic and Interventional Radiology, University Hospital Würzburg, Oberdürrbacher Str. 6, 97080 Würzburg, Germany

**Keywords:** CT angiography, Aorta, Photon-counting detector CT, Radiation dose reduction, Contrast agent reduction, Spectral imaging

## Abstract

Our objective was to assess dedicated low-dose (LD) and low-contrast (LC) scan protocols for aortic angiography, compared to standard protocols in photon-counting CT (PCD-CT) and energy-integrating CT (EID-CT). 57 patients (23 women) who underwent aortic PCD-CT angiography (Nov. 23 – July 24) were assessed retrospectively. 19 received a low-contrast protocol (LC; 14 mg iodine, image quality level IQ64), 18 a low-dose protocol (LD; 21 mg iodine, IQ42), and 20 a combined low-dose/low-contrast protocol (LD/LC) according to defined patient characteristics. In addition, two groups of 20 BMI-matched patients were created, one of which underwent CTA using the standard PCD-CT protocol and the other using EID-CT, and these were compared with the new protocols. CNR was calculated. Overall quality, luminal contrast, vessel sharpness, and artifacts were rated by four radiologists. LD enabled ~ 40% dose-reduction compared to EID-CT (3.3 ± 0.8 versus 5.3 ± 0.4mGy; *p* < 0.01) but higher CNR (33.5 ± 6.1 vs. 25.1 ± 6.4; *p* < 0.01). CNR of LC (26.3 ± 5.6) and LD/LC (25.6 ± 9.1) did not differ significantly to EID-CT (25.1 ± 6.4; *p* > 0.99). Image quality of LC was deemed superior to EID-CT (5 [5–5] vs. 4 [4–4]; *p* < 0.01). LD (4 [4–5]) and LD/LC (4 [4–4]) demonstrated comparable image quality to EID-CT (*p* = 0.52/0.94). In conclusion even the LD/LC protocol provided image quality comparable to EID-CT despite substantial reduction of radiation and contrast agent dose. Special dose-reduced and contrast-reduced protocols are feasible without compromising diagnostic image quality.

**Supplementary Information:**

The online version contains supplementary material available at https://doi.org/10.1007/s10554-026-03757-y.

## Introduction

CT angiography (CTA) plays an essential role in the diagnosis and follow-up of a variety of cardiovascular diseases. Despite advances in diagnostics and therapy, aortic diseases, in particular, are associated with substantial mortality and a high burden of disease [1] requiring repeated CTA both pre- and post-therapeutically [2]. These examinations mainly pose two risks: On the one hand, the repeated application of ionizing radiation, especially for young patients, with the potential to induce malignant diseases [3]. On the other hand, the potential of contrast-induced nephropathy due to repeated use of iodine contrast media, especially in patients with pre-existing renal insufficiency [4].

Photon-counting detector (PCD) CT has the potential to reduce both. PCD directly converts incoming X-ray photons into electrical signals and enables measurement of their individual energy. The data obtained contains spectral image information without the need for a dedicated pre-selected scan mode allowing for the elimination of image noise by setting a low keV detection threshold, offering the potential for radiation dose reduction compared to energy-integrating detector (EID) CT [5].

A previous study performed by Hennes et al. demonstrated a markedly higher contrast-to-noise ratio (CNR) in PCD-CTA compared to EID-CTA [6]. The CNR advantage of about 33% could be directly translated into reduced radiation dose as a quasi-linear relationship between applied radiation dose and CNR has previously been demonstrated [7]. Likewise, a previous study by Higashigaito et al. suggested a linear relationship between contrast agent dose and achievable CNR [8]. According to the results of this study, a 33% reduction of contrast agent dose should be realizable, while ensuring the attainment of diagnostic image quality.

Clinically indicated PCD-CTA protocols with reduced contrast agent dose, reduced radiation dose, and a combination of both were evaluated for quality management reasons. Therefore, we compared the image quality with standard CTA protocols in both PCD-CT and EID-CT.

## Materials and methods

### Patient population

This retrospective single-center study was conducted according to the principles of the declaration of Helsinki. The need for additional written consent was waived by the local ethics committee who granted permission to conduct the study (protocol number: 20241024 01).

Patients who underwent PCD-CTA of the thoracoabdominal aorta with one of three patient characteristics adapted protocols between November 2023 and July 2024 were included in the retrospective study. Criteria that led to study exclusion were deviations from the respective scan or contrast medium protocol, body-mass-index (BMI) > 35 kg/m^2^, and incomplete examination volumes. 20 BMI-matched patients who underwent PCD-CTA with the standard manufacturer recommended protocol (SSe) between January 2021 and October 2023 as well as EID-CTA between February 2020 and July 2023 were included as reference standard.

### CTA protocols and patient assignment

CT examinations were performed on a first-generation cadmium-telluride-based PCD-CT (Naeotom Alpha, Siemens Healthineers, Forchheim, Germany) using ECG-triggered high-pitch examinations in the cranio-caudal direction (pitch factor 3.2, rotation time 0.25 s). Multienergetic data sets (Quantum plus) were generated in all patients with a fixed tube voltage of 120 kVp, standard resolution scan mode with 2 × 2 pixel binning, and a collimation of 144 × 0.4 mm. At our institution, one of three specific CTA scan protocols is employed based on patients’ age and clinical status:

- A protocol with reduced radiation dose using a 33% lower image quality level (IQ 42 instead of IQ 64 in the standard protocol) with an iodine delivery rate (IDR) of 1.23 gI/s (LD).
- A protocol with reduced contrast medium dosage using 40 ml instead of 60 ml of iodinated contrast medium (Imeron 350 mg iodine/ml; Bracco, Milan, Italy), resulting in a reduction of the iodine amount from 21 g to 14 g per CTA scan and an IDR of 0.88 gI/s (LC).
- A combination of both with reduced radiation dose and contrast medium application with an IDR of 0.88 gI/s (LD/LC).

The clinical criteria used to allocate patients to their clinical examination protocols were age, BMI and creatinine level. Patients aged ≤ 50 years or > 50 years with normal weight (BMI < 25 kg/m^2^) were assigned to the LD/LC protocol. In patients > 50 years of age who were also overweight (BMI between 25.0 and 29.9 kg/m^2^) or obese grade I (BMI between 30.0 and 34.9 kg/m^2^), creatinine levels were the deciding criterion. Patients with creatinine levels ≤ 1 mg/dl were assigned to the low-dose protocol (LD), while patients with creatinine levels > 1 mg/dl were assigned to the low-contrast protocol (LC). Patients with iodine allergy (after prophylactic therapy) and hyperthyroidism (under blockade) were exceptions and were generally assigned to the LC protocol (Table 1). 

**Table 1:** Clinical criteria used for patients’ assignment to examination protocols

LC	LD	LD/LC
		Age ≤ 50 years
Age > 50 years and BMI 25.0–34.9 kg/m^2^		Age > 50 years and BMI 18.5–24.9 kg/m^2^
Creatinine > 1.0 mg/dl	Creatinine ≤ 1.0 mg/dl	Creatinine independent
(Suspected) iodine allergy or hyperthyroidism		

LC, reduced contrast agent dose protocol; LD, reduced radiation dose protocol; LD/LC, combination protocol with both reduced radiation dose and contrast agent dose; BMI, Body mass index

The established PCD-CTA protocol was performed with identical parameters as the new protocols but with IQ 64 and a contrast medium dose of 60 ml (SSe). The standard EID-CTA protocol was performed on a third-generation dual-source CT scanner (Somatom Force, Siemens Healthineers) in single-energy mode with automatic tube voltage selection (CARE kV, Siemens Healthineers), whereby 100 kVp with a reference tube current of 350 mAs was selected for all examinations. By using both detectors, each with a collimation of 96 mm × 0.6 mm and a flying focal spot, an effective collimation of 192 mm × 0.6 mm was realized. Both SSe and EID-CT had an IDR of 1.23 gI/s.

### Contrast agent protocol

The contrast agent was applied as follows: The standard contrast protocol for reduced radiation dose protocols consisted of a 30 ml NaCl pre-bolus, followed by 60 ml contrast medium and a 30 ml NaCl post-bolus, all at a flow rate of 3.5 ml/s. For the contrast medium reduced protocols, the pre-bolus and post-bolus were reduced to 20 ml NaCl and only 40 ml contrast agent was administered at a flow rate of 2.5 ml/s. Application was performed via a 20-gauge peripheral venous cannula and an automatic injector (CT motion, Ulrich GmbH, Ulm, Germany). To ensure optimal arterial contrast, bolus tracking was used with a region of interest (ROI) positioned in the descending thoracic aorta with a trigger threshold of 100 HU and a delay of 7 s.

### Image reconstruction

CTA were reformatted as axial image stacks with a slice thickness / increment of 3 mm using a soft vascular convolution kernel (Bv36) and medium iterative reconstruction strength (PCD-CTA: QIR level 3; EID-CTA: ADMIRE 3, both Siemens Healthineers). PCD-CTA were reconstructed as virtual monoenergetic images (VMI) at 55 keV as recommended by the manufacturer. The field of view was set to be 368 × 368 mm with adjustments depending on the abdominal girth. The pixel matrix for EID-CTA was fixed at 512^2^ while the matrix of PCD-CTA was either 512^2^ or 768^2^ pixels, depending on the field of view.

### Quantitative image comparison

To determine the attenuation within the arterial vessels (HU_artery_), ROI were placed at five sites in the ascending aorta (1), aortic arch (2), descending thoracic aorta (3), abdominal aorta (4), and common iliac artery (5). ROI were drawn as large as possible excluding any near-wall artifacts. On the same image slice, additional ROI (30 mm^2^) were placed to measure the attenuation in muscle tissue (HU_muscle_) and the standard deviation of the HU values in adipose tissue (SD_fat_). The latter was defined as the measure of image noise. CNR was calculated using the following formula:

$$\:CNR=\frac{\left({HU}_{artery}-{HU}_{muscle}\right)}{{SD}_{fat}}$$

### Subjective image comparison

The subjective image quality was independently rated by four radiologists (two residents with 2 years and two board-certified radiologists with 7 and 9 years of experience in cardiovascular imaging) blinded for the image protocols using picture archiving and communication system with preset hangings. All readers were blinded to clinical information and protocol parameters. Furthermore, the image order was randomized for each reader and readers were asked to take breaks of at least 3 days after each 20 images they rated to reduce reader bias. The evaluation criteria were overall image quality, luminal attenuation, vessel sharpness and absence of blooming or beam hardening artifacts using a 5-point rating scale (5 – excellent; 4 – good; 3 – moderate; 2 – poor; 1 – non diagnostic).

### Statistical analysis

Considering the results of this groups previous study [6] where a very high power of d = 1.8 was measured. Assuming the same power for this study would result in an estimated sample size of *n* = 8. As the parameters of the previous study cannot be applied one-to-one to the current study, we assume a more conservative, still high power of d = 1. This results in a required group size of *n* = 19. The dataset was analyzed using specialized software (DATAtab e.U., Graz, Austria). Normal distribution was tested by Kolmogorov Smirnov tests. Metric-scaled values with normal distribution are reported as mean ± standard deviation, while non-parametric data are stated as median values with interquartile ranges (IQR). To analyze objective image quality parameters, parametric ANOVA without repeated measures were employed using corrected Bonferroni to control the Type I error rate. Linear regression models were used to determine to assess the influence of Patient BMI on CTDI_Vol_, mean intraluminal attenuation and CNR. For analysis of subjective image quality, the ratings of all observers were pooled and compared using non-parametric Kruskal-Wallis tests. Statistical significance is assumed at *p* < 0.05. Kendall’s W was calculated to assess interrater reliability. Coefficients of < 0.5 were considered indicative of moderate agreement, between 0.5 and 0.8 of good agreement, and > 0.8 of excellent agreement .

## Results

### Patient sample and characteristics

**Fig. 1:**
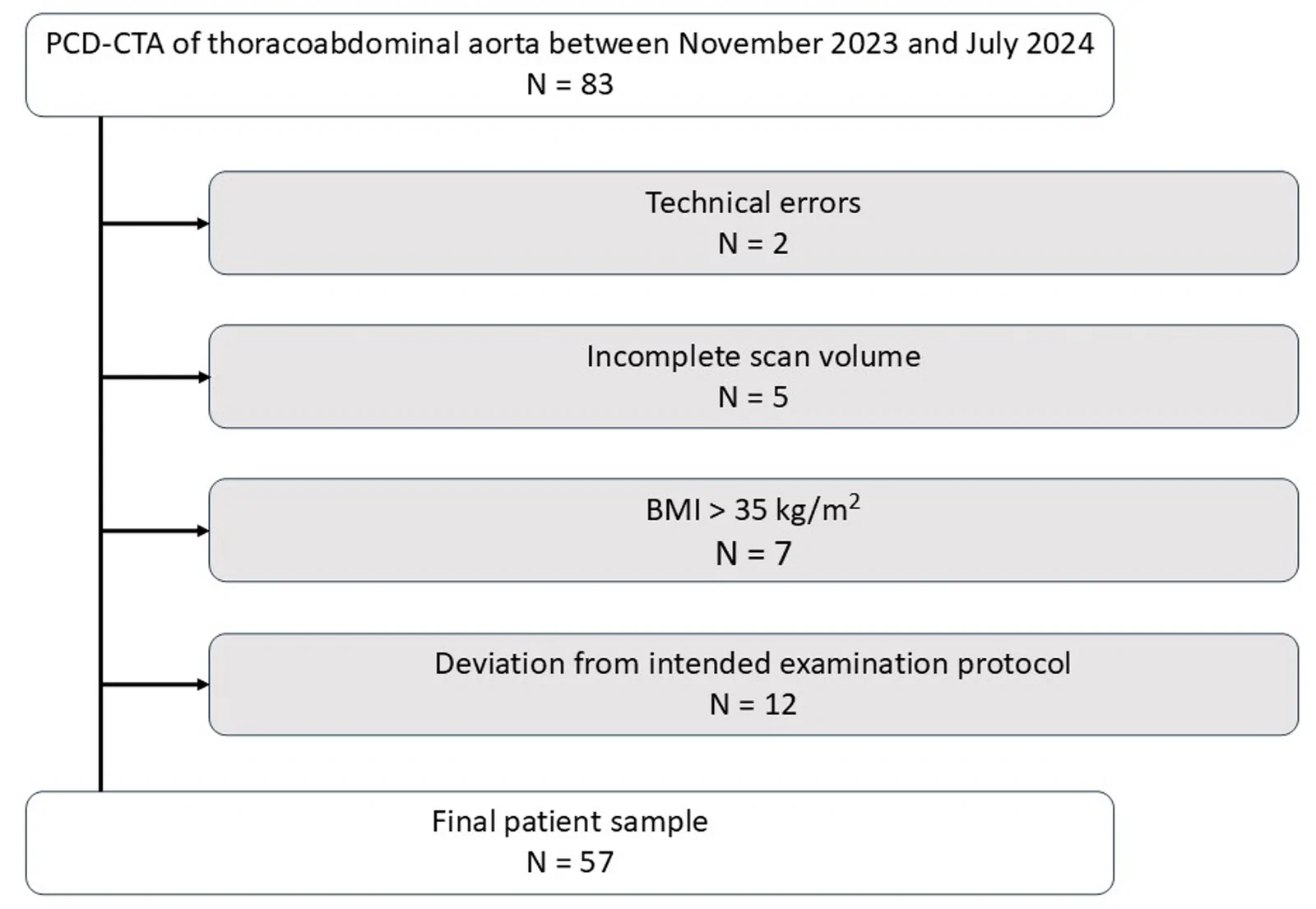
Flowchart visualizing patient exclusion criteria and the final sample of the study cohort. PCD-CTA, photon-counting detector computed tomography angiography; BMI, body mass index

During the study period, 83 patients underwent PCD-CTA of the thoracoabdominal aorta. Of these, two were excluded due to technical errors, twelve due to deviations from the examination protocol, five due to incomplete scan volumes and seven due to a BMI > 35 kg/m^2^. The final patient sample consisted of 57 examinations, of which 19 were performed with LC (7 female), 18 with LD (4 female) and 20 with LD/LC (8 female), as summarized in Fig. 1. In addition, 20 patients who had undergone both PCD-CTA and EID-CT using standard protocols were included and individually BMI-matched to the LD subgroup with the smallest possible deviation. There was no significant difference in patient age between the three study groups (LD: 69.2 ± 9.6; LC: 65.3 ± 7.9years; LD/LC: 59.9 ± 16.0; *p* ≥ 0.14). The age of patients from the SSe and EID-CTA samples was 67.6 ± 8.9 and 63.6 ± 12.4 years, respectively, with no significant difference to the study subgroups (*p* ≥ 0.36). Patients in the LD/LC group had the lowest BMI according to the assignment criteria (24.3 ± 3.4 kg/m^2^; *p* < 0.01), while no significant difference was ascertained for patients receiving the LD and LC protocols (28.3 ± 2.8 vs. 28.8 ± 3.8 kg/m^2^; *p* > 0.99). The BMI of patients in the reference SSe (27.1 ± 2.0 kg/m^2^) and EID-CT groups (26.9 ± 1.9 kg/m^2^) was comparable to LD and LC (*p* ≥ 0.41), but higher than LC/LD (*p* ≤ 0.04). A summary of patient characteristics is provided in Table 2. All examinations were performed as elective imaging; of these, 3 were preoperative, and 93 were follow-ups (65 aortic surgery; 12 aortic aneurysms; 16 Stanford type B aortic dissections). A detailed breakdown by subgroup is provided as Table S1 of the supplementary material. 

**Table 2:** Patient characteristics

Parameter	LC	LD	LD/LC	SSe	EID-CT
Age (years)	65.3 ± 6.5	69.22 ± 8.2	59.9 ± 13.7	67.6 ± 7.5	63.6 ± 8.4
Weight (kg)	82.8 ± 11.7	85.44 ± 13.5	71.9 ± 13.8	83.8 ± 7.7	79.5 ± 8.1
Height (m)	1.7 ± 0.1	1.7 ± 0.1	1.7 ± 0.1	1.8 ± 0.1	1.7 ± 0.1
BMI (kg/m^2^)	28.3 ± 2.1	28.5 ± 3	24.3 ± 3.4	27.1 ± 1.7	26.9 ± 1.7
CTDI_Vol_ (mGy)	4.8 ± 0.8	3.3 ± 0.7	2.8 ± 0.7	4.5 ± 0.6	5.3 ± 0.3
SSDE (mGy)	5.6 ± 0.7	3.8 ± 0.6	3.4 ± 0.5	5.3 ± 0.5	6.4 ± 0.6
DLP (mGy*cm)	339.4 ± 67.2	212.4 ± 54.1	200.9 ± 53.8	292.0 ± 44.1	349.1 ± 37.7

Values are given as mean ± standard deviation

LC, reduced contrast agent dose protocol; LD, reduced radiation dose protocol; LD/LC, combination protocol with both reduced radiation dose and contrast agent dose; SSe, photon-counting detector CT with standard settings; EID-CT, energy-integrating detector CT

BMI, body mass index; CTDI_Vol_, volume CT dose index; SSDE, size specific dose estimate; DLP, dose length product

### Radiation dose

Both low radiation dose protocols (LD 3.3 ± 0.8 mGy; LD/LC 2.8 ± 0.8 mGy) showed lower volume CT dose indices (CTDI_Vol_) than SSe (4.5 ± 0.7 mGy; *p* < 0.01), EID-CTA (5.3 ± 0.4 mGy; both *p* < 0.01), and the LC protocol (4.8 ± 1.0 mGy; both *p* < 0.01). No significant difference was observed between LD and LD/LC (*p* = 0.25) as well as between LC and SSe (*p* > 0.99) and between LC and EID-CTA (*p* > 0.99). The radiation exposure associated with each protocol is given in Table 3. 

**Table 3:** Objective image quality parameters

Parameter	LC	LD	LD/LC	SSe	EID-CT
Luminal attenuation (HU)	387.6 ± 85.8	501.9 ± 75.5	388.2 ± 107.0	478.0 ± 95.0	350.4 ± 64.4
Attenuation muscle (HU)	52.6 ± 2.8	52.9 ± 2.0	56.5 ± 2.3	45.6 ± 4.8	54.1 ± 4.3
Image noise (HU)	12.7 ± 0.7	13.5 ± 0.8	13.1 ± 0.5	12.9 ± 1.3	12.0 ± 1.2
Contrast-to-noise-ratio	26.3 ± 5.6	33.5 ± 6.1	25.6 ± 9.1	34.0 ± 7.9	25.1 ± 6.4

Values are given as mean ± standard deviation

LC, reduced contrast agent dose protocol; LD, reduced radiation dose protocol; LD/LC, combination protocol with both reduced radiation dose and contrast agent dose; SSe, photon-counting detector CT with standard settings; EID-CT, energy-integrating detector CT

HU, Hounsfield unit; SD, standard deviation

### Objective image quality

**Fig. 2:**
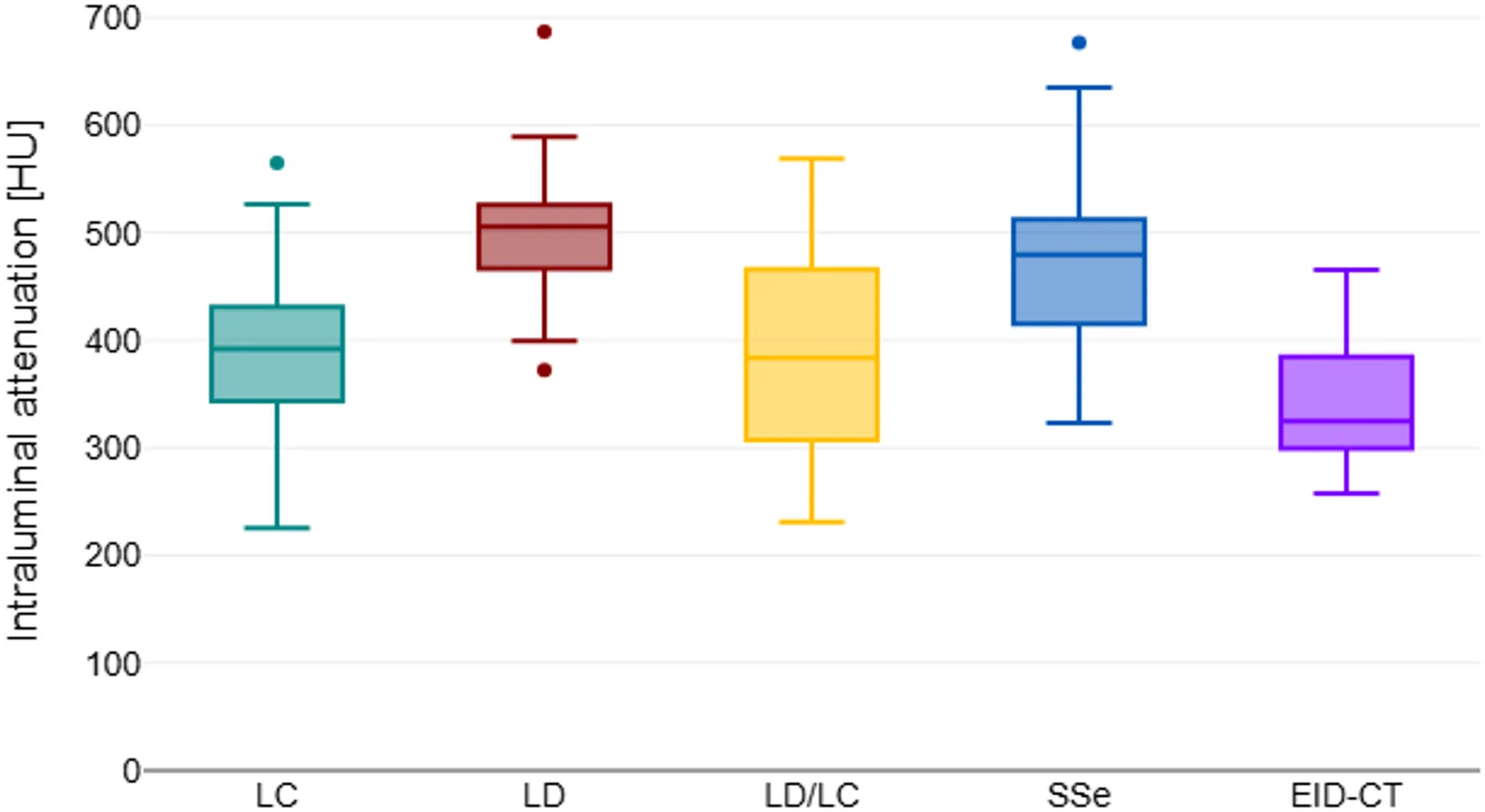
Box and whisker plot diagram summarizing the intraluminal attenuation achieved by the used CT protocols. Boxplots show median and 25%/75% quartile (solid line), minimum and maximum as well as mean value with standard deviation (dashed line). Photon-counting-detector CT (PCD-CT) with standard settings (SSe), energy-integrating-detector CT (EID-CT), PCD-CT with reduced radiation dose (LD), PCD-CT with reduced contrast agent dose (LC) and PCD-CT with a combination of reduced radiation dose and contrast agent dose (LD/LC). HU, Hounsfield units

The luminal attenuation of EID-CTA (350.4 ± 64.4 HU) was the lowest of the five groups including the contrast agent dose-reduced PCD-CT protocols (LC 387.6 ± 85.8 HU; LD/LC 388.2 ± 107.0 HU; each *p* > 0.99). In contrast, the PCD-CTA protocols with the same contrast agent dose as EID-CTA generated higher intraluminal attenuation (LD & SSe vs. LC, LD/LC and EID-CT; *p* < 0.01) without a significant difference between each other (SSe 478.0 ± 95.0 HU; LD 501.9 ± 75.5 HU; *p* > 0.99). The measured values of intraluminal attenuation are summarized in Table 3 and are illustrated in Fig. 2. 

**Fig. 3:**
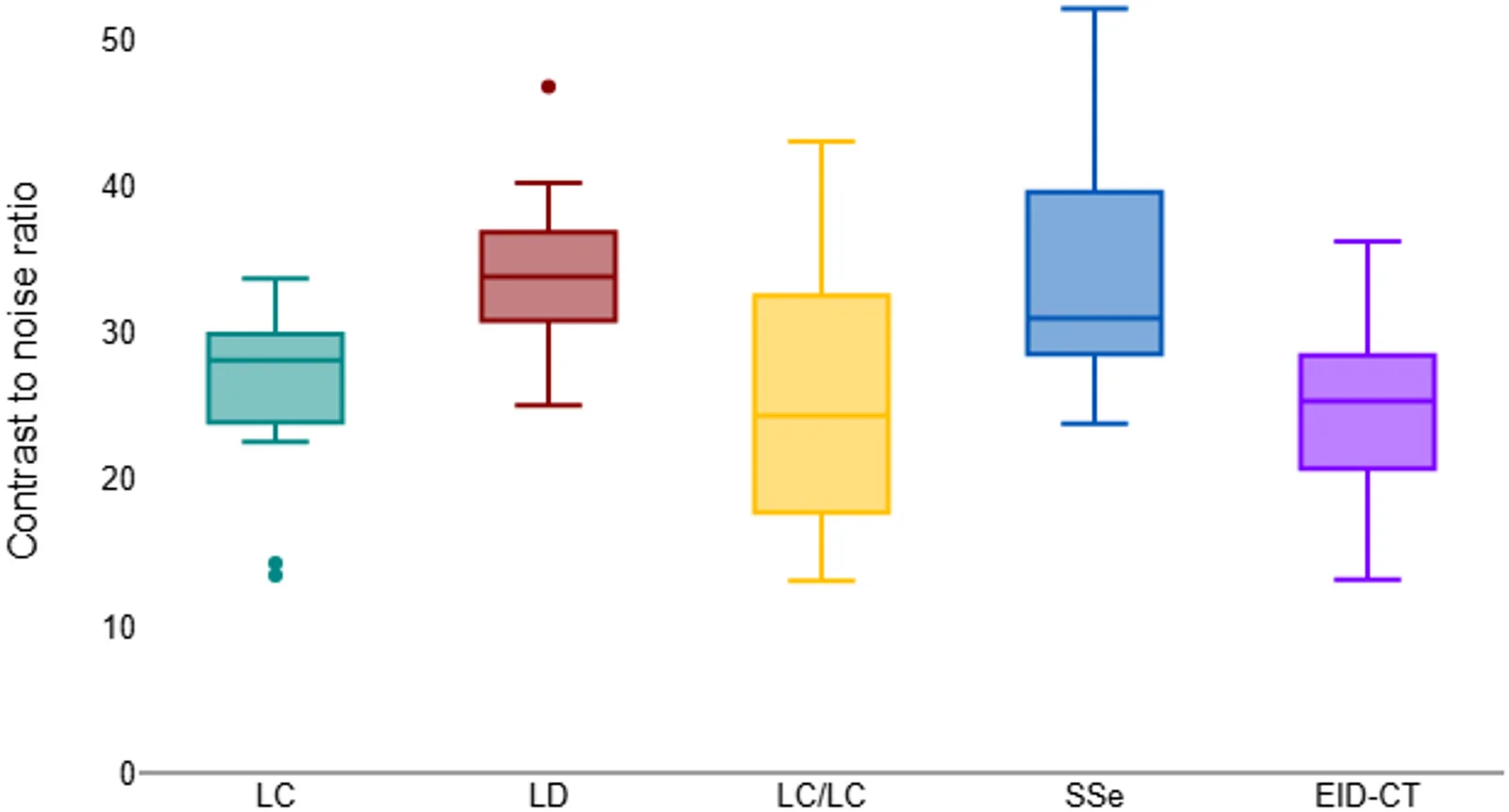
Box and whisker plot diagram summarizing the contrast-to-noise ratio achieved by the used CT protocols. Photon-counting-detector CT (PCD-CT) with standard settings (SSe), energy-integrating-detector CT (EID-CT), PCD-CT with reduced radiation dose (LD), PCD-CT with reduced contrast agent dose (LC) and PCD-CT with a combination of reduced radiation dose and contrast agent dose (LD/LC)

**Fig. 4:**
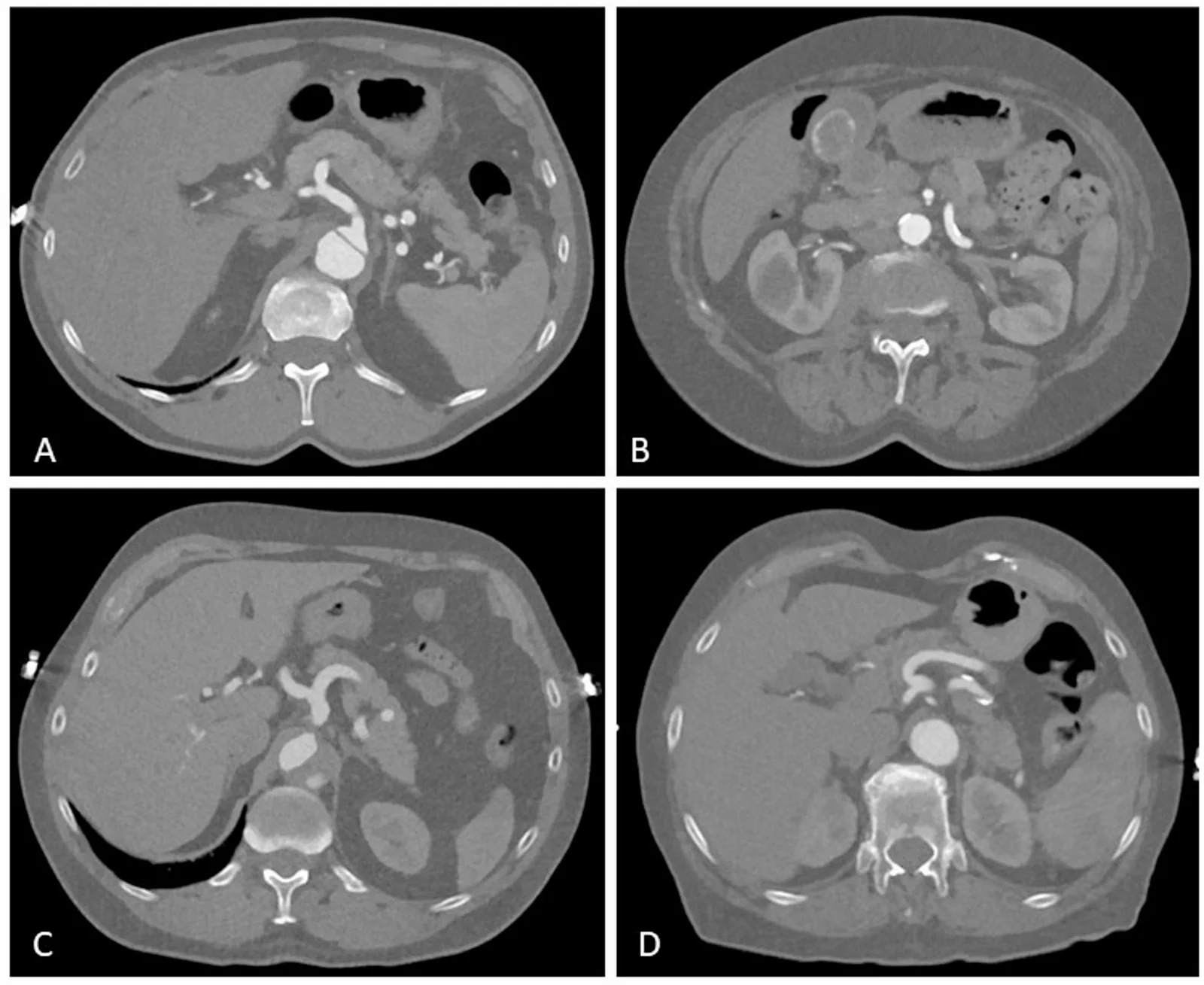
Representative image quality of the CT protocols. CT angiography in different patients with comparable body mass index generated with EID-CT (**A**) and PCD-CT with reduced radiation dose (**B**), reduced contrast agent dose (**C**) and a combination of both (**D**)

Consequently, the lowest average CNR was calculated for EID-CTA (25.1 ± 6.4), followed by LD/LC (25.6 ± 9.1) and LC (26.3 ± 5.6) without significant difference between them (*p* > 0.99, each). Despite both being adapted based on the CNR achieved by the EID-CT LD (33.5 ± 6.1) achieved a higher CNR than LC (26.3 ± 5.6) (*p* = 0.03). The LC protocol achieved CNR values comparable to EID-CTA (*p* > 0.99) but lower than those of SSe (34.0 ± 7.9; *p* = 0.01). LD on the other hand was superior to EID-CT (*p* < 0.01) and comparable with SSe (*p* > 0.99). CNR of LD and SSe were higher than of the LD/LC protocol (*p* ≤ 0.01). CNR values are summarized in Table 3 and illustrated in Fig. 3. Exemplary axial CTA images of the respective protocols are shown in Fig. 4. A linear relationship between radiation dose and BMI was demonstrated for all PCD-CT protocols, but not for EID-CT. The investigation revealed no discernible correlation between BMI and intraluminal contrast or CNR. A concise synopsis of the regression analysis can be found in Table S2 and S3 of the supplementary material. 

### Subjective image quality

**Fig. 5:**
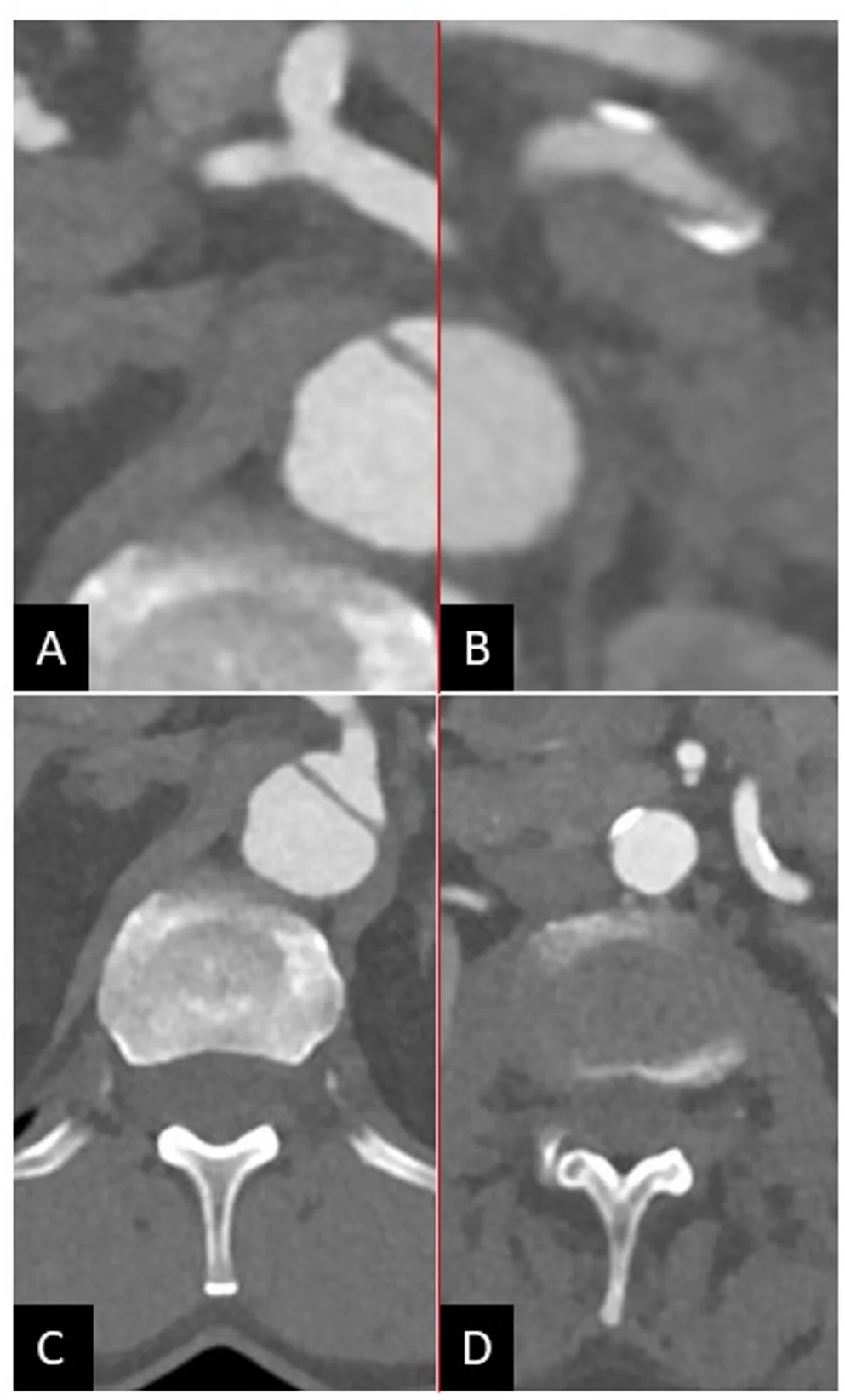
Pairwise image comparison to illustrate differences in vascular sharpness and image noise. Direct comparison to emphasize the similar vessel attenuation of EID-CT (**A**) and contrast agent reduced PCD-CT (**D**) or rather image noise of EID-CT (**C**) and of radiation dose reduced PCD-CT (**D**)

All adapted protocols achieved good to excellent ratings in the categories image quality and luminal attenuation. The LC protocol received better ratings for image quality (median 5 [IQR 5–5]) than LD (4 [4–5]; *p* < 0.01) and LD/LC (4 [4–4]; *p* < 0.01), while there was no significant difference between LD and LD/LC (*p* = 0.22). In contrast, the luminal attenuation of LD (5 [5–5]) was preferred to LC (4 [3–5]; *p* < 0.01) and LD/LC (4 [4–5]; *p* = 0.02) without significant difference between the latter (*p* = 0.19). Regarding the categories vessel sharpness and blooming/hardening, no significant differences were ascertained between the investigated protocols (*p* ≥ 0.2). Exemplary CTA images are shown in Fig. 5. 

Comparing the subjective image quality results of the study protocols and EID-CTA, only LC provided superior image quality (5 [5–5] vs. 4 [4–4]; *p* < 0.01). Regarding luminal attenuation, only LD was better than EID-CT (5 [5–5] vs. 4 [4–5]; *p* = 0.02). The image quality of SSe was superior to LD and LD/LC (5 [5–5] vs. 4 [4–5] and 4 [4–4]; *p* ≤ 0.01) but not to LC (5 [5–5]; *p* = 0.82). Regarding luminal attenuation, SSe (5 [5–5]) was rated better than LC (4 [3–5]; *p* < 0.01) and LD/LC (4 [4–5]; *p* = 0.01), but not LD (5 [5–5]; *p* = 0.95). While the luminal attenuation in LD was deemed superior compared with EID-CTA (4 [4–5]; *p* = 0.02), there was no significant difference compared to LC (4 [3–5]; *p* = 0.2) and LD/LC (4 [4–5]; *p* = 0.99).

**Fig. 6:**
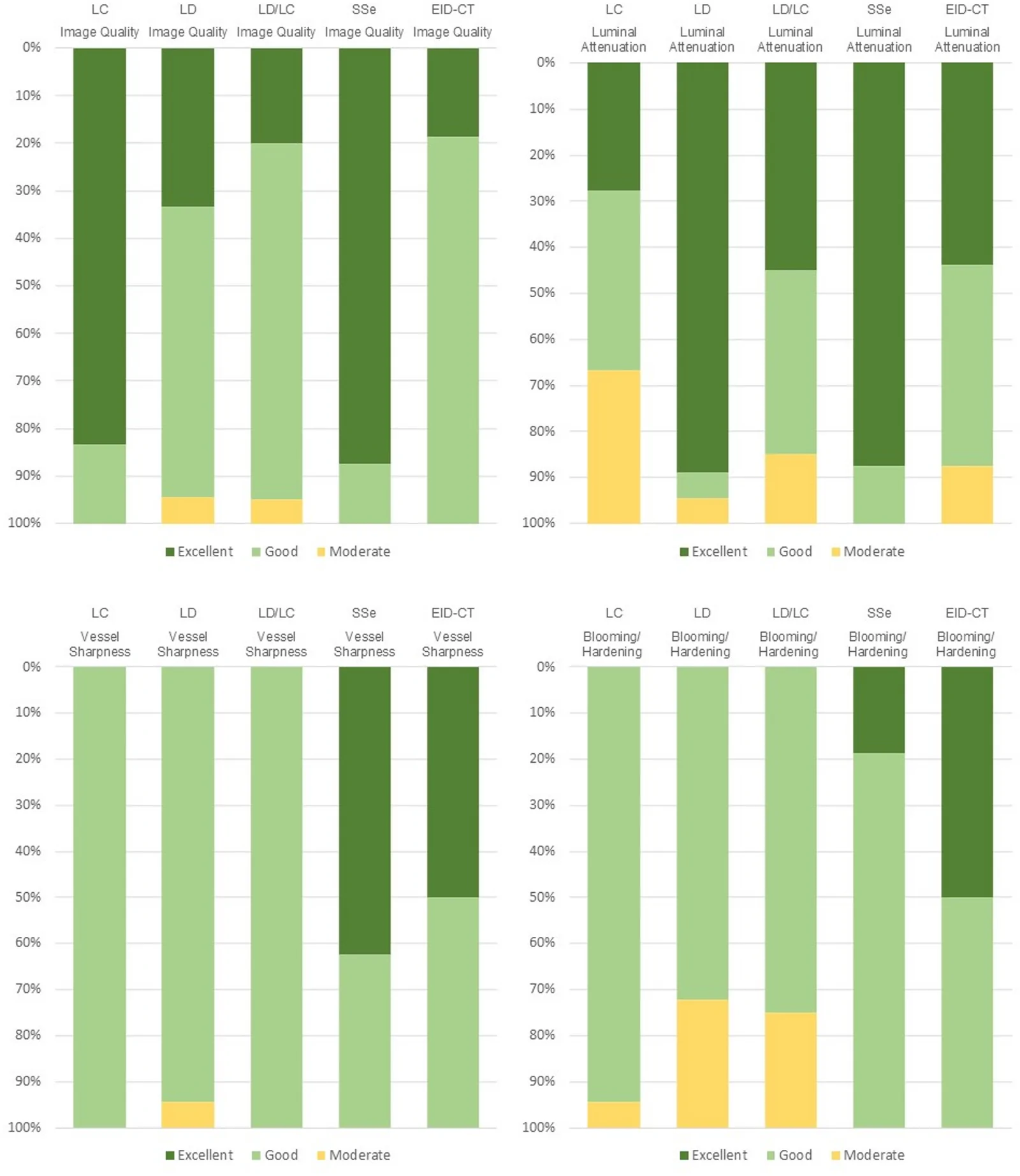
Bar charts provide an overview of the subjective image quality ratings. Subjective image quality in the low contrast agent dose protocol (LC), low radiation dose protocol (LD), the combination protocol (LD/LC), photon-counting detector CT with standard settings (SSe) and energy-integrating detector CT (EID-CT). The assessed criteria were overall image quality (top left), luminal attenuation (top right), vessel sharpness (bottom left) and the influence of blooming/beam hardening artifacts (bottom right)

Regarding vessel sharpness and blooming/beam hardening, both SSe and EID-CTA were considered better than the three study protocols (*p* ≤ 0.01), with the exception of LC versus SSe for artifacts (*p* = 0.16). Table 4 provides a summary of the raters’ pooled assessments; the results are also illustrated in Fig. 6. 

**Table 4:** Subjective image quality parameters

Parameter	LC	LD	LD/LC	SSe	EID-CT
Image quality	5 [5–5]	4 [4–5]	4 [4–4]	5 [5–5]	4 [4–5]
Luminal attenuation	4 [3–5]	5 [5–5]	4 [4–5]	5 [5–5]	4 [4–5]
Vessel sharpness	4 [4–4]	4 [4–4]	4 [4–4]	5 [4–5]	4 [4–5]
Blooming/beam hardening artifacts	4 [4–4]	4 [3–4]	4 [3–4]	4 [4–4]	4 [4–5]

Values are given as median and interquartile ratio (IQR)

LC, reduced contrast agent dose protocol; LD, reduced radiation dose protocol; LD/LC, combination protocol with both reduced radiation dose and contrast agent dose; SSe, photon-counting detector CT with standard settings; EID-CT, energy-integrating detector CT

Interrater reliability was good to excellent for all categories (Kendall’s W > 0.8) except for vessel sharpness with moderate interrater agreement (Kendall’s W = 0.4). 

## Discussion

The aim of this retrospective study was to evaluate photon-counting CT angiography protocols with reduced contrast agent dose, reduced radiation dose, and a combined reduction of both, compared with standard protocols on photon-counting and energy-integrating detector (EID) CT systems. The principal finding of this study is that clinically adapted PCD-CTA protocols allow for a substantial reduction of either contrast agent dose or radiation dose without compromising diagnostic image quality compared to third-generation EID-CTA. The combined low-dose/low-contrast (LD/LC) protocol demonstrated comparable objective and subjective image quality metrics to EID-CTA, despite a reduction of approximately 40% in radiation dose and 30% in iodine load.

In particular, the low-contrast (LC) protocol enabled a one third reduction in iodine dose, while maintaining both CNR and subjective image quality at a level comparable to full-contrast EID-CTA. This finding is clinically relevant for patients at risk of contrast-induced nephropathy and underscores the potential of PCD technology to mitigate contrast-related risks in aortic CTA, while even further reduction of the amount of iodine should achieve sufficient vascular contrast [9]. Further studies are needed to investigate this assumption.

In contrast, the assumed linear correlation between radiation dose and CNR [7] could not be confirmed. the low-dose (LD) protocol achieved a relevant reduction in radiation exposure without deterioration of overall image quality with largely consistent CNR values, supporting the use of PCD-CTA in radiation-sensitive patient populations. These results suggest the possibility of further dose reduction. However, it must be noted that a measurable effect on subjective image quality was observed, in contrast to the objective values. This limitation should be taken into account when attempting further dose reductions and countermeasures such as lowering the keV.

Despite comparable objective image quality, reduced-dose PCD-CTA protocols showed slightly inferior subjective ratings for vessel sharpness and blooming or beam-hardening artifacts compared with standard protocols. This effect is likely attributable to the lower radiation dose and the use of low-keV VMI reconstructions, which enhance iodine contrast at the expense of increased image noise. Nevertheless, these limitations did not impair overall diagnostic image quality. The objective image quality of all three customized protocols was higher than the results in the EID-CTA sample. It must be noted that the intrinsic spectral information in PCD-CTA datasets was used for post-processing and reconstruction of VMI at 55 keV, as recommended by the manufacturer. Since spectral imaging is not possible with the EID-CT used in combination with ECG-triggered high-pitch CT angiography, as this requires the width of both detectors [10] and may result in dose disadvantages [11, 12], instead a low-kV scan protocol with 90/100 kVp was used instead for improved iodine contrast [13]. Such technical differences in the scanners used should be considered as potential confounders as well.

A number of research has previously demonstrated a relationship between BMI and radiation dose. However, the precise nature of this association is contingent upon factors such as scanner technology, acquisition protocols, and dose modulation algorithms [10]. In the present study, a linear relationship between BMI and CTDI_Vol_ was observed for all PCD-CT protocols, whereas no such association was found in the EID-CTA cohort. Potential explanations for the observed differences may be found in the automatic exposure control strategies and dose modulation behaviors exhibited by the two scanner generations [5]. Additionally, the fixed tube voltages may necessitate higher doses for adipose patients, particularly in the context of EID-CT scans [14].

Despite comparable objective image quality, reduced-dose PCD-CTA protocols showed slightly inferior subjective ratings for vessel sharpness and blooming or beam-hardening artifacts compared with standard protocols as expectable [15].This effect is likely attributable to the lower radiation dose and the use of low-keV virtual monoenergetic image (VMI) reconstructions, which enhance iodine contrast at the expense of increased image noise [16]. A number of studies have been published on this topic; however, these studies have frequently focused on only a few keV levels. The range of recommendations extends from 40 keV to 65 keV; however, higher radiation doses (CTDI_Vol_ of at least 1 mGy higher) result in a concomitant increase in image noise [17, 18]. Consequently, this study adhered to the conservative average value of 55 keV. Not least, distortions due to the use of a 5-point rating scale come into question, since suchlike scales are susceptible to a variety of biases [19, 20]. Nevertheless, these limitations did not impair overall diagnostic image quality.

In our study, all datasets were reconstructed using a soft kernel (Bv36). However, previous research has demonstrated that the choice of reconstruction kernel significantly influences both vessel sharpness and overall image quality [21, 22]. Sharper kernels, in particular, have been shown to improve the sharpness of vessels and the differentiation between calcifications and the contrasted lumen [23].

Patient selection played a key role in protocol assignment. The combined LD/LC protocol was intentionally restricted to patients with lower BMI to avoid excessive image noise and potential non-diagnostic examinations [24, 25]. Consequently, the applicability of this protocol to overweight or obese patients remains uncertain and should be addressed in future studies.

Several methodological limitations should be considered. Firstly, although this retrospective single-centre study included a statistically sufficient patient cohort based on prior effect size calculations, the results require confirmation in larger multicentre studies. Second, allocation to clinically indicated protocols may have introduced selection bias. To improve comparability despite the small group sizes, patients with a BMI > 35 kg/m² were excluded, which limits the generalisability of the results to obese patients. Secondly, only scanner hardware from one vendor was evaluated, which may restrict the transferability of the results to other systems. Thirdly, only one VMI level (55 keV) and one reconstruction setting were analyzed, and optimizing these parameters warrants further investigation. Fourthly, due to technical constraints, high-pitch spirals with EID-CTA could only be acquired without additional spectral information. Finally, the lack of randomization may have caused selection bias, and subtle differences in image appearance between PCD-CTA and EID-CTA may have introduced recognition bias in the subjective assessment of image quality.

## Conclusion

Low-dose and low-contrast protocols for aortic PCD-CTA deliver good to very good objective image and subjective image quality metrics. Even when combining radiation dose reduction by 40% and contrast medium reduction by 30% in one protocol, the resulting images are not inferior compared to third-generation EID-CT.

## Supplementary Information

Below is the link to the electronic supplementary material.

Supplementary Material 1

## Data Availability

The datasets generated and/or analyzed during the current study are not publicly available but are available from the corresponding author on reasonable request.

## Abbreviations

CNR: Contrast-to-noise ratio
CTA: Computed tomography angiography
EID: Energy-integrating detector
IQ: Image quality level
LC: Low-dose protocol
LD: Low-contrast protocol
PCD: Photon-counting detector
ROI: Region of interest
SSe: Standard settings
VMI: Virtual monoenergetic images
