# Supplementary material for: Retrospective assessment of low-dose and low-contrast agent protocols for photon-counting CT angiography of the aorta
Source: Int J Cardiovasc Imaging. 2026 Jun 19;42(10):1891–901. doi: 10.1007/s10554-026-03757-y (PMC13645889; doi:10.1007/s10554-026-03757-y)
Supplement: Supplementary file 1 [file 10554_2026_3757_MOESM1_ESM.docx]

**Supplementary Table 1:**

**Indications for CT-angiographies by protocol**

Reduced contrast agent dose (LC):

- 13 follow-up examinations after aortic surgery.
- 2 preoperative examinations prior to cardiothoracic surgery.
- 3 follow-up examinations of Stanford Type B aortic dissections.

Reduced radiation dose (LD):

- 13 follow-up examinations after aortic surgery.
- 1 preoperative examination prior to cardiothoracic surgery.
- 2 follow-up examinations of Stanford Type B aortic dissections.
- 2 follow-up examinations for aortic aneurysms.

Combination (LD/LC):

- 14 follow-up examinations after aortic surgery.
- 2 preoperative examinations prior to cardiothoracic or vascular surgery.
- 2 follow-up examinations of Stanford Type B aortic dissections.
- 2 follow-up examinations for aortic aneurysms.

Standard settings (SSe):

- 14 follow-up examinations after aortic surgery.
- 2 follow-up examinations for aortic aneurysms.
- 4 follow-up examinations for Stanford Type B aortic dissections.

Energy integrating detector CT (EID-CT):

- 15 follow-up examinations after aortic surgery.
- 1 follow-up examination of an aortic aneurysm.
- 4 follow-up examinations of Stanford type B aortic dissections.

**Supplementary Table 2:**

**Linear correlation between BMI and CTDI_Vol_**

| Cohort | R | R2 | p | Formula |
| --- | --- | --- | --- | --- |
| LC | 0.85 | 0.7 | < 0.001 | CTDI_Vol_ = -0.06 + 0.06 × BMI |
| LD | 0.66 | 0.4 | 0.002 | CTDI_Vol_ = 0.50 + 0.03 × BMI |
| LC/LC | 0.86 | 0.66 | < 0.001 | CTDI_Vol_ = 0.23 + 0.04 × BMI |
| SC | 0.53 | 0.24 | 0.015 | CTDI_Vol_ = 1.25 + 0.04 × BMI |
| EID-CT | 0.37 | 0.09 | 0.107 | CTDI_Vol_ = 4.12 + 0.02 × BMI |

LC, reduced contrast agent dose protocol; LD, reduced radiation dose protocol; LD/LC, combination protocol with both reduced radiation dose and contrast agent dose; SSe, photon-counting detector CT with standard settings; EID-CT, energy-integrating detector CT; BMI, body mass index; CTDI_Vol_, volume CT dose index.

**Supplementary Table 3:**

**Linear correlation between BMI and Mean intraluminal attenuation (MIA)**

| Cohort | R | R2 | p | Formula |
| --- | --- | --- | --- | --- |
| LC | 0.11 | 0.01 | 0.642 | MIA = 486.51 - 3.50 × BMI |
| LD | 0.18 | 0.03 | 0.469 | MIA = 607.50 - 3.48 × BMI |
| LC/LC | 0.34 | 0.11 | 0.144 | MIA = 648.75 - 10.74 × BMI |
| SC | 0.22 | 0.05 | 0.356 | MIA = 757.53 - 10.30 × BMI |
| EID-CT | 0.11 | 0.01 | 0.643 | MIA = 449.87 - 3.69 × BMI |

LC, reduced contrast agent dose protocol; LD, reduced radiation dose protocol; LD/LC, combination protocol with both reduced radiation dose and contrast agent dose; SSe, photon-counting detector CT with standard settings; EID-CT, energy-integrating detector CT; BMI, body mass index.

**Supplementary Table 4:**

**Linear correlation between BMI and Contrast-to-noise-ratio (CNR)**

| Cohort | R | R2 | p | Formula |
| --- | --- | --- | --- | --- |
| LC | 0.14 | 0.02 | 0.579 | CNR = 33.92 – 0.27 × BMI |
| LD | 0.31 | 0.09 | 0.215 | CNR = 46.53 – 0.44 × BMI |
| LC/LC | 0.39 | 0.16 | 0.084 | CNR = 52.43 – 1.10 × BMI |
| SC | 0.24 | 0.06 | 0.297 | CNR = 60.17 – 0.96 × BMI |
| EID-CT | 0.15 | 0.02 | 0.518 | CNR = 38.82 – 0.51 × BMI |

LC, reduced contrast agent dose protocol; LD, reduced radiation dose protocol; LD/LC, combination protocol with both reduced radiation dose and contrast agent dose; SSe, photon-counting detector CT with standard settings; EID-CT, energy-integrating detector CT; BMI, body mass index.
